# Characteristics of gut microbiota in patients with metabolic associated fatty liver disease

**DOI:** 10.1038/s41598-023-37163-4

**Published:** 2023-06-20

**Authors:** Chao Yang, Jianguo Xu, Xiaomin Xu, Wen Xu, Bangzhuo Tong, Shulin Wang, Rujie Ji, Yan Tan, Ying Zhu

**Affiliations:** 1 Department of Gastroenterology, Shenzhen Hospital of Southern Medical University, Shenzhen, Guangdong China; 2 Department of Liver Disease Center, Shenzhen Hospital of Southern Medical University, Shenzhen, Guangdong China; 3 Xbiome Co. Ltd., Shenzhen, Guangdong China

**Keywords:** Microbiology, Diseases

## Abstract

Metabolic associated fatty liver disease (MAFLD) is rising in incidence and is an increasingly common cause of cirrhosis and hepatocellular carcinoma (HCC). Alterations in the gut microbiota have been shown to correlate with the development and progression of MAFLD. However, little is known regarding differences in the gut microbiomes of MAFLD patients and healthy cohorts, and subgroups at the abnormal activity of hepatic enzymes in China. In this study, we enrolled 81 MAFLD patients and 25 healthy volunteers. The fecal microbiota was assessed using 16S rRNA gene sequencing and metagenomic sequencing. The results suggested that *Ruminococcus obeum* and *Alistipes* were most enriched in healthy individuals when compared with MAFLD patients. Microbe‐set Enrichment Analysis (MSEA) results showed *Dorea*, *Lactobacillus* and *Megasphaera* are enriched in MAFLD group. We also found that *Alistipes* has negatively related to serum glucose (GLU), gamma-glutamyl transferase (GGT), and alanine aminotransferase (ALT). Moreover, the abundance of *Dorea* was found to be significantly overrepresented in the MAFLD patients and the degree of enrichment increased with the increasing abnormal liver enzyme. An increase in *Dorea*, combined with decreases in *Alistipes* appears to be characteristic of MAFLD patients. Further study of microbiota may provide a novel insight into the pathogenesis of MAFLD as well as a novel treatment strategy.

## Introduction

Metabolic associated fatty liver disease (MAFLD, previously known as Nonalcohol fatty liver disease, NAFLD) is classified as a wide spectrum of liver diseases, ranging from hepatic steatosis and steatohepatitis to fibrosis and cirrhosis^1,2^. The disease is characterized by fat accumulation in hepatocytes exceeding 5% of the liver’s weight in the absence of excessive alcohol consumption and other stimulating factors, such as drugs and virus^3^. MAFLD has a high general prevalence of 22–29% among adults worldwide, greater than 75% of whom are overweight and obese^4^. Despite the recent refinement of the diagnostics and management of MAFLD, including liver biopsy, clinical indicators, and therapeutic intervention, the patients still endure a long and challenging illness course, such as long-suffering relapse, immunological rejection, and metabolic syndrome^5^.

The pathological mechanism concerning the onset and progression of MAFLD is complicated and has not been fully elucidated. Widely accepted the key risk factors include genetic and epigenetic factors, dietary determinants, insulin resistance, hepatocellular lipotoxicity, pro-inflammatory factors, and gut microbiome^6^. Approximately 10^^14^ microbial cells reside within the gut microbiota, making it a virtual metabolic and endocrine organ^7^. The intestinal microbiota plays a key role in metabolic diseases, such as obesity, fatty liver, and diabetes^8,9^. The liver of patients with MAFLD are frequently affected by the adverse effects from the altered gut microbiome through the gut–liver axis^10^. Through the portal vein, pro-inflammatory and other factors derived from intestinal microbiota and immune response products enter the hepatic tissue^11^. Consuming a diet high in saturated fats and calories over time may cause dysbiosis in the gut microbiota. In turn, this leads to imbalanced bile acid pools and intestinal barrier dysfunction, and then an increase in the translocation of bacteria and metabolites and pro-inflammatory components from bacteria entering into the liver. In general, dysfunction of the gut–liver axis caused by bacterial proliferation in the intestine, intestinal dysbiosis, and alteration of the intestinal permeability have a significant influence on the development and progression of MAFLD^12^. There is considerable evidence that the gut microbiome can induce obesity, insulin resistance and liver steatosis. It has been accepted that changing the composition of gut microbiota can regulate obesity development^13^. And then the gut microbiome has recently come under intense scrutiny and has been used as a therapeutic tool in combating MAFLD^14^.

To demonstrate gut microbiota signatures, researchers have compared the gut microbiota of patients with MAFLD, NASH, and cirrhosis with those of individuals with a healthy liver^15^. Besides, in MAFLD, MAFLD fibrosis, and cirrhosis, gut microbiome signatures could be used as noninvasive diagnostic biomarkers^9^. Other studies have shown a lower diversity of microbiota in patients with NAFLD compared to healthy subjects^16,17^. A meta-analysis of patients with NAFLD showed abnormalities in gut microbiota composition, including high levels of *Escherichia*, *Prevotella*, and *Streptococcus* and low abundance of *Coprococcus*, *Feacalibacterium*, and *Ruminococcus* in patient stools with NAFLD, suggesting abnormal gut microbiota composition^18^. As of now, rifaximin, prebiotics, and probiotics are the most promising treatments for patients with MAFLD. Although indirect effects have been observed, this area is still undergoing development. Individuals respond differently to interventions should be noted. Precise characterization of broad microbial microbiota changes in differential subgroup of MAFLD patients is therefore necessary, in order to unravel the relevance of microbiota interventions in the management of MAFLD.

Many studies in the global community have focused on the connection between the gut microbiome and MAFLD in recent years. However, there is limited knowledge of the differences in the gut microbiomes of MAFLD patients and healthy cohorts in China. In this study, we enrolled 81 MAFLD individuals and 25 healthy volunteers for the analysis of the microbial characteristics in MAFLD patients and subgroups of MAFLD patients to provide a rational basis for development of microbiota interventions for MAFLD based on the fecal microbiome.

## Results

### The clinical and physical variables

Table 1 presents the general characteristics of the 106 included participants. The mean age was approximately 36 years in both healthy and MAFLD groups. Of the 106 participants, 81 (68 men, 13 women) were in the MAFLD group. Body mass index (BMI) level was significantly lower in the healthy volunteer group compared with the MAFLD group. Table 2 presents the general information of the MAFLD subgroups, with or without liver enzyme abnormality. No significant difference was observed in BMI between the two subgroups (P = 0.057).

**Table 1:** Baseline demographic data summary of all participants.

Variable	Healthy, N = 25^1^	MAFLD, N = 81^1^	p-value^2^ (adjust. p value^3^)
Age	35 (11)	37 (10)	0.2 (0.35)
Gender			0.8 (0.90)
Female	5/25 (20%)	13/81 (16%)	
Male	20/25 (80%)	68/81 (84%)	
Ethnic			> 0.9 (0.90)
Han	25/25 (100%)	79/81 (98%)	
Man	0/25 (0%)	2/81 (2.5%)	
Height (cm)	170 (7)	169 (7)	0.8 (0.90)
Weight (kg)	60 (7)	79 (11)	< 0.001 (0.00)
BMI (kg/m^2^)	20.7 (1.2)	27.3 (3.1)	< 0.001 (0.00)
Waistline (cm)	74 (7)	99 (10)	< 0.001 (0.00)

^1^Mean (SD), n/N (%).

^2^Wilcoxon rank sum test; Fisher’s exact test.

^3^p-values were adjusted by FDR method.

**Table 2:** Baseline demographic data summary of MAFLD patients.

Variable	Liver enzyme abnormal, N = 34^1^	Liver enzyme normal, N = 28^1^	p-value^2^ (adjust. p value^3^)
Age	35 (8)	37 (8)	0.4 (0.60)
Gender			> 0.9 (0.90)
Female	6/34 (18%)	5/28 (18%)	
Male	28/34 (82%)	23/28 (82%)	
Ethnic			> 0.9 (0.90)
Han	33/34 (97%)	27/28 (96%)	
Man	1/34 (2.9%)	1/28 (3.6%)	
Height (cm)	169 (7)	169 (8)	0.8 (0.90)
Weight (kg)	80 (11)	76 (10)	0.2 (0.33)
BMI (kg/m^2^)	27.92 (3.24)	26.59 (2.33)	0.057 (0.15)
Waistline (cm)	100 (10)	97 (9)	0.2 (0.33)
Lipids			
LDL (mmol/L)	3.30 (1.01)	3.45 (0.77)	0.62 (0.74)
CHOL (mmol/L)	5.06 (1.15)	4.84 (1.17)	0.55 (0.71)
TG (mmol/L)	2.89 (2.95)	1.75 (1.15)	0.02 (0.06)
GLU (mmol/L)	5.36 (0.59)	5.00 (0.49)	0.01 (0.04)
HDL (mmol/L)	1.04 (0.20)	1.14 (0.23)	0.10 (0.20)
Hepatic fibrosis			
CAP (dB/m)	312.71 (27.26)	288.96 (37.95)	0.01 (0.04)
LSM (kPa)	7.35 (2.52)	5.32 (1.80)	0.00 (0.00)
PIIINP (ng/mL)	13.38 (7.50)	8.74 (3.42)	0.00 (0.00)
CIV (ng/mL)	43.96 (22.04)	32.18 (10.65)	0.02 (0.06)
LN (ng/mL)	71.89 (23.31)	61.78 (19.66)	0.08 (0.18)
HA (ng/mL)	45.95 (21.64)	45.22 (12.27)	0.52 (0.71)

^1^Mean (SD), n/N (%).

^2^Wilcoxon rank sum test; Fisher’s exact test.

^3^p-values were adjusted by FDR method.

**Figure 1:**
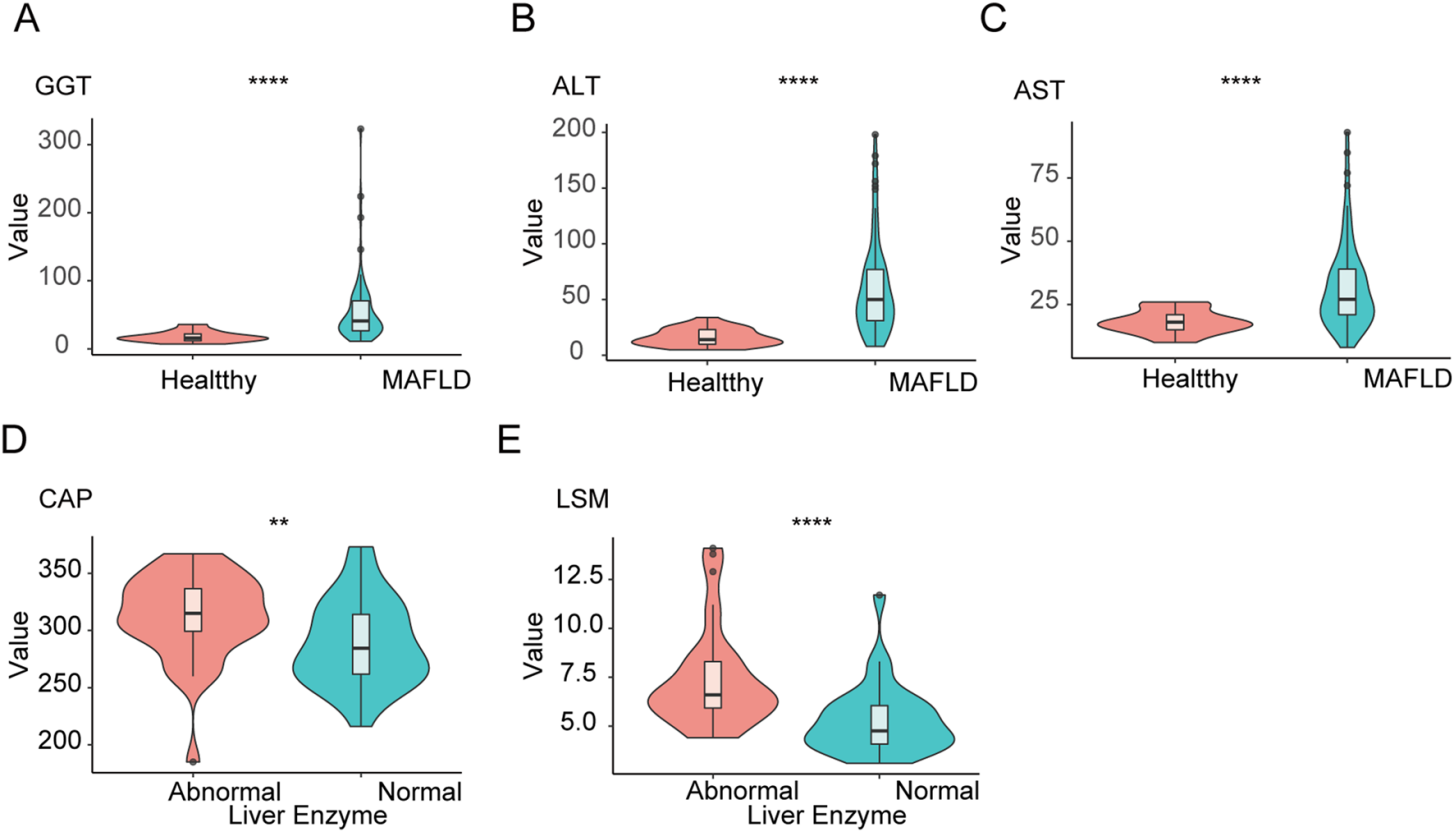
The clinicopathological indicators involved in patients. (**A-C**) The boxplot shows GGT, ALT and AST of healthy and MAFLD subjects. (**D-E**) Significantly different clinical parameters are presented in MAFLD subgroups with or without liver enzyme abnormal. **P < 0.01, ****P < 0.0001.

We next analyzed the differences in the physical variables between the two groups. The results shown that the levels of gamma-glutamyl transferase (GGT; Fig. 1A), alanine aminotransferase (ALT; Fig. 1B), and aspartate aminotransferase (AST; Fig. 1C) were significantly higher in subjects with MAFLD. From the analysis of MAFLD subgroups, we also found that the levels of clinical variables including controlled attenuation parameter (CAP; Fig. 1D), liver stiffness measurement (LSM; Fig. 1E), fasting insulin, hypersensitive C-reactive protein, lactate dehydrogenase (LDH), triglyceride (TG), glucose (GLU), type III procollagen peptide N terminal (PIIINP), and collagen type IV (C-IV) were significantly higher in MAFLD subjects with abnormal liver enzyme but not in subjects with normal liver enzyme (Table 2).

### Analysis of gut microbiota using metagenomic data in species level

**Figure 2:**
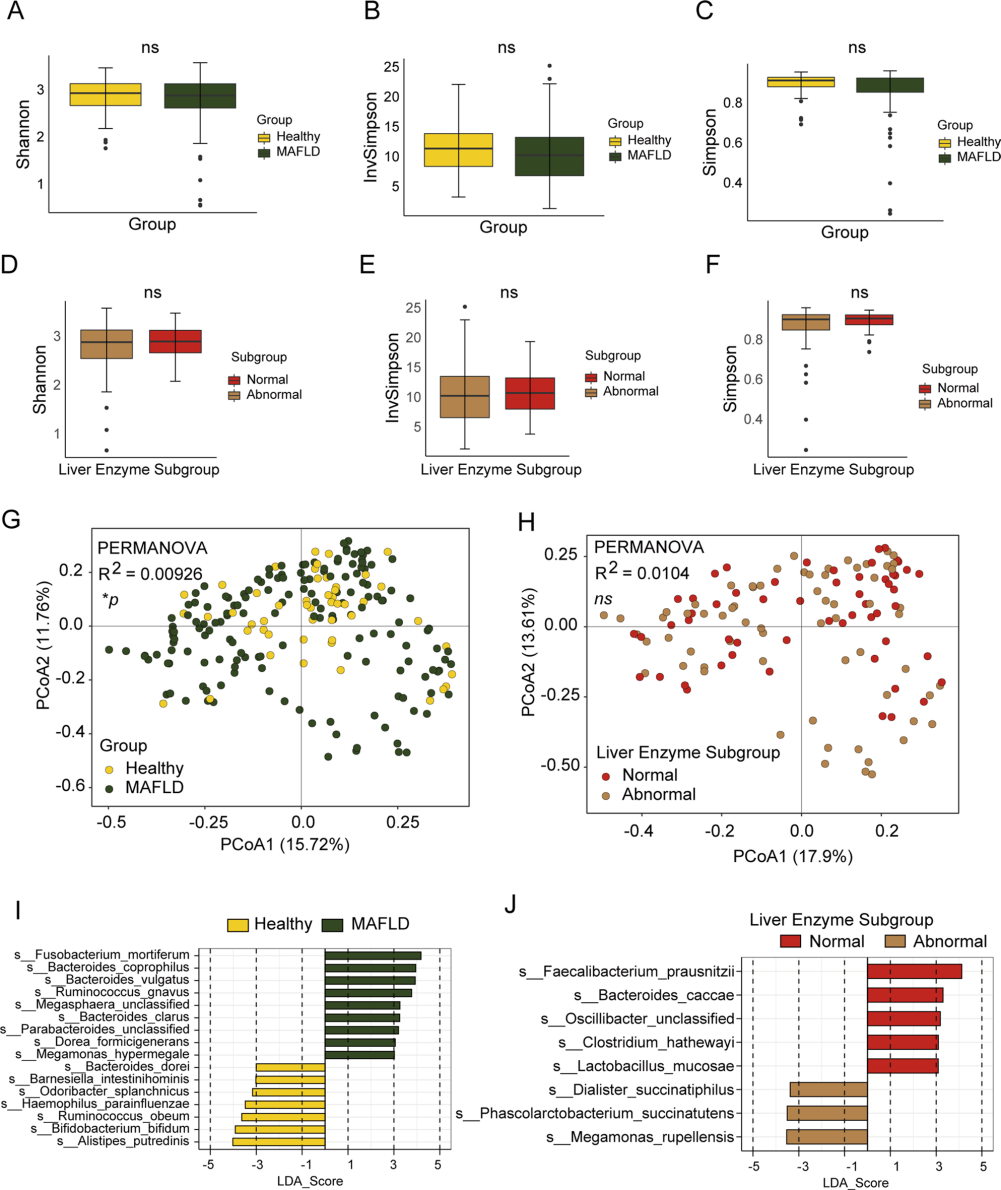
Diversity of the gut microbiome characteristics base on metagenomic sequencing in species level. (**A–C**) Alpha diversity indices of the intestinal bacterial communities of healthy control and MAFLD group. (**D–F**) Alpha diversity indices of the intestinal bacterial communities of different MAFLD subgroups. Shannon, InvSimpson, and Simpson indices. (**G**,**H**) Different letters above the bars indicate significant differences as determined by PERMANOVA. Samples are identified by filled circles. In both PCoA1 and PCoA2 principal components (PCoA1 and PCo2) are plotted. The percentage of variance in the dataset explained by each axis is reported. (**I**,**J**) LefSe analysis showed predominant gut microbiota. *P < 0.05, ns *P* > 0.05.

To gain insight into microbiome diversity, Shannon, Simpson, and InvSimpson diversity indices were determined for alpha diversity analysis of the metagenome data. The Shannon Simpson, and InvSimpson diversity indexes indicated that no significant difference of microbiome diversity was observed in the healthy volunteers and MAFLD groups (Fig. 2A–C). Similarly, no significant difference existed among MAFLD subjects with abnormal liver enzyme and with normal liver enzyme (Fig. 2D,E). But we observed a tendency of increase of the microbiome diversity in the health volunteer group and MAFLD subjects with abnormal liver enzyme.

Principal coordinate analysis provided an overview of the gut microbiome and reflected the β-diversities of the different groups. The β-diversity was clearly higher in the healthy volunteer group than that in the MAFLD group (Fig. 2G). However, the β-diversity showed no difference among the subgroups of MAFLD subjects with abnormal and normal liver enzyme (Fig. 2H). To identify the predominant gut microbiota in difference group, LEfSe analysis was performed. The results showed that there were a number of different genera of gut microbiota between the healthy and the MAFLD groups, and a trend could be observed that in the MAFLD groups the relative richness of *Bacteroides vulgatus* was much higher than that in the healthy group. Moreover, *Ruminococcus Obeum* and *Alistipes putredinis* were highly enriched in the healthy group (Fig. 2I). We also found that *Bacteroides caccae* were highly enriched in normal liver enzyme group (Fig. 2J).

### Analysis of gut microbiota using 16S data in genus level

**Figure 3:**
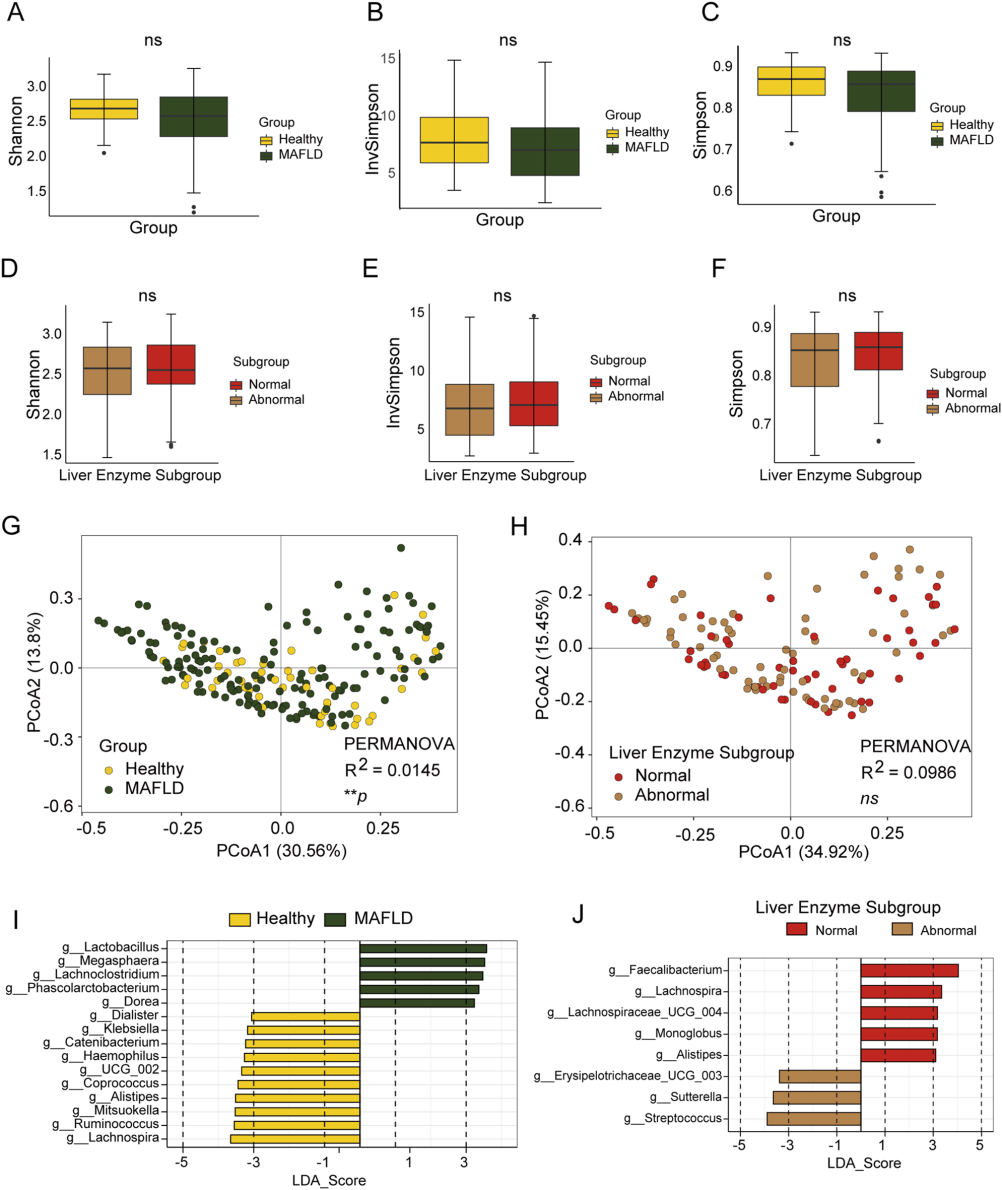
Diversity of the gut microbiome characteristics base on 16S data in genus level. (**A–C**) Alpha diversity indices of the intestinal bacterial communities of healthy control and MAFLD group. (**D–F**) Alpha diversity indices of the intestinal bacterial communities of different MAFLD subgroups. Shannon, InvSimpson, and Simpson indices. (**G**,**H**) Different letters above the bars indicate significant differences as determined by PERMANOVA. Samples are identified by filled circles. In both PCoA1 and PCoA2 principal components (PCoA1 and PCoA2) are plotted. The percentage of variance in the dataset explained by each axis is reported. (**I**,**J**) LefSe analysis showed predominant gut microbiota. **P < 0.01, ns *P* > 0.05.

Microbial 16S rRNA gene amplicons were denoised into amplicon sequence variants (ASVs) to provide a high-resolution^19,20^ view of how MAFLD impacted the community structure. To uncover the microbiota distribution and genera in MAFLD and healthy groups, fecal samples were performed 16S rRNA sequencing and Shannon, Simpson, and InvSimpson indexes calculated. No diversities of microbiome were significantly different between MAFLD and healthy group (Fig. 3A–C). Subgroups were derived according to different liver enzyme for further analysis. The result shown that the microbiome diversities did not differ significantly between subgroups (Fig. 3D–F).

To display similarities of microbiome compositions between difference group, β-diversity was calculated using UniFrac method, and PCoA was performed. The results presented a significantly different distribution among healthy and MAFLD groups using permutational multivariate analysis of variance analysis (PERMANOVA) (Fig. 3G). But did not differ between MAFLD subgroups (Fig. 3H). These results suggest that the MAFLD group had significant different gut microbiome compositions from healthy group. Meanwhile, with the aid of the LEfSe methods, we conducted analysis of different species between groups and identified a series of bio- markers as shown in the Fig. 3I,J. The resulting cladogram presents the structure of the gut microbiome and the predominant bacteria in the healthy and MAFLD groups. Notably, results of LEfse analysis also showed that *Alistipes* higher enriched in MAFLD subgroup of liver enzyme normal compared with liver enzyme abnormal subgroup. *Alistipes* belongs to the family *Rikenellaceae*, which was also decreased in NAFLD patients based on a study by Zhu, L. et al.^21^. And several studies have linked the presence of *Alistipes* genus with a healthy state^22^. Therefore, the restored intestinal *Alistipes* communities contributed to the ameliorated MAFLD.

### Microbe‐set enrichment analysis (MSEA) of gut microbiota

**Figure 4:**
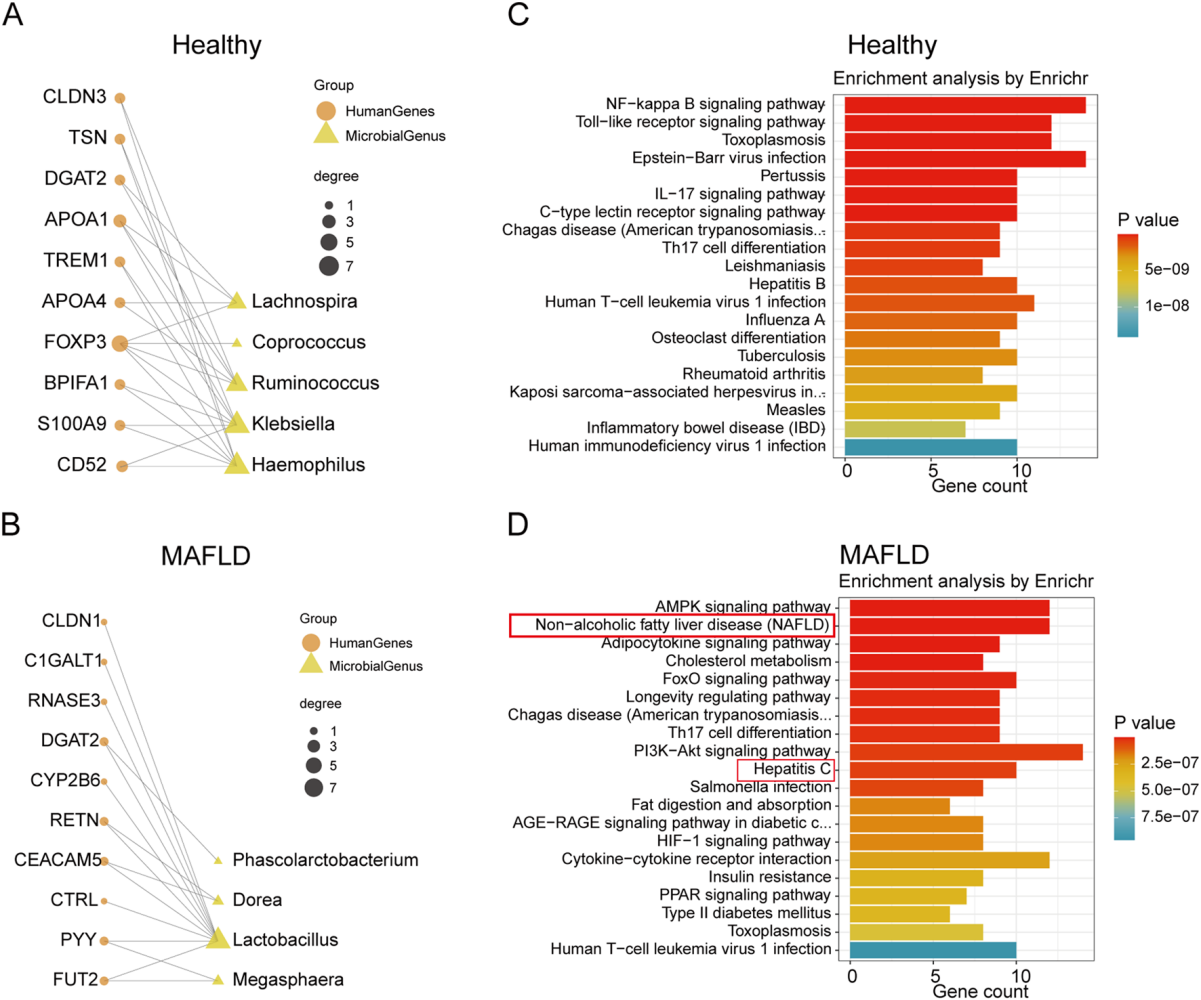
Microbe‐set enrichment analysis (MSEA) of fecal microbiota. (**A-B**) Bipartite graph visualizing the enriched mammalian genes with their associated gut microbes in healthy controls versus MAFLD. Genes are charted as orange round nodes whereas gut microbes are plotted as yellow square. The sizes of the nodes are proportional to the number of edges in the bipartite graph whereas the width of the edges indicates the strength of the enrichment measured by combined scores from the MSEA algorithm. (**C-D**) Network of top enriched human KEGG pathways for genes enriched from MSEA analysis.

MSEA analysis^23^ can be incorporated with microbiome profiling pipelines to determine the mechanisms underlying host‐microbiome interactions. Then, we analyzed the 16S microbiome profiling data with a focus on characterizing the functions of microbes with differential abundance (DA) in MAFLD compared to healthy controls using MSEA. We prioritized human genes enriched for those MAFLD-related DA microbes. Among the top enriched genes, we found that several interesting microbe-related-gene associations (Fig. 4A,B). For example, *Dorea, Lactobacillus, Megasphaera* are enriched in MAFLD group. We next performed enrichment analysis for the top genes that are enriched from the DA microbes in MAFLD compared to healthy controls. The results showed that most genes were enriched in MAFLD and Hepatitis C pathway in MAFLD group (Fig. 4C,D).

### KEGG orthology metabolic pathway analysis

**Figure 5:**
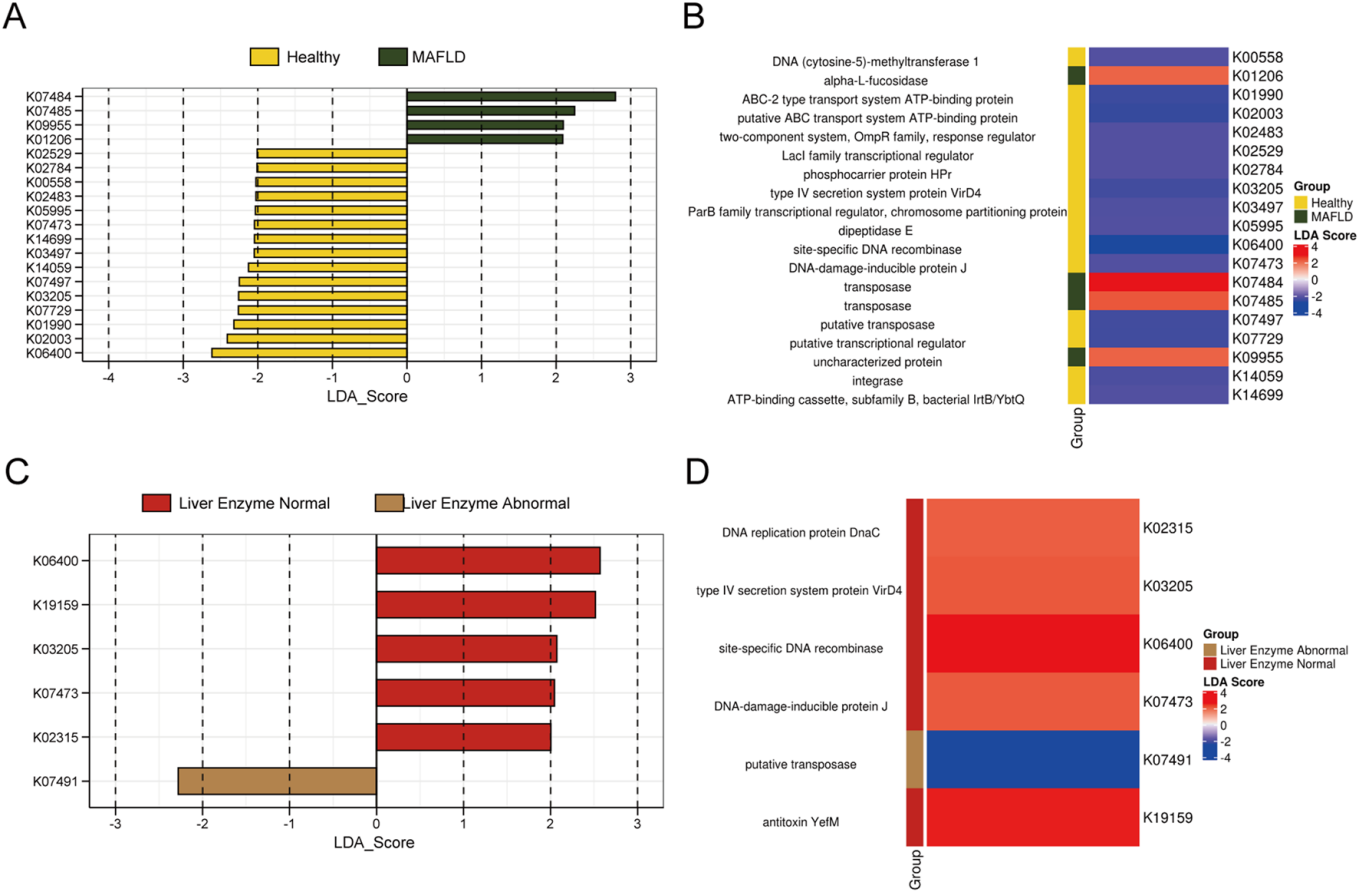
KEGG orthology metabolic pathway analysis. (**A-B**) LDA scores and Heatmap showed significant pathway differences between the healthy and MAFLD groups. (**C-D**) LDA scores showed significant pathway differences between the MAFLD subgroups with or without liver enzyme abnormal.

Functional analysis was conducted with HUMAnN2^24^ (version 2.8.1) by aligning clean reads (reads after removing host contamination) to KEGG protein database (version 2020.7). Furthermore, mapped KEGG genes were enriched to KO entries by MinPath^25^. LEfSe analysis was performed to identify potentially enriched KEGG Orthology. We found that K07484 (transposase) and K07485 (transposase) were enriched in the MAFLD group (Fig. 5A,B). K06400 (site-specific DNA recombinase), and K19159 (antitoxin YefM) were enriched in the MAFLD with normal liver enzyme, but K07491 (REP-associated tyrosine transposase) was enriched in the MAFLD with abnormal liver enzyme (Fig. 5C,D).

### Associations between metagenomics data and clinical parameters

**Figure 6:**
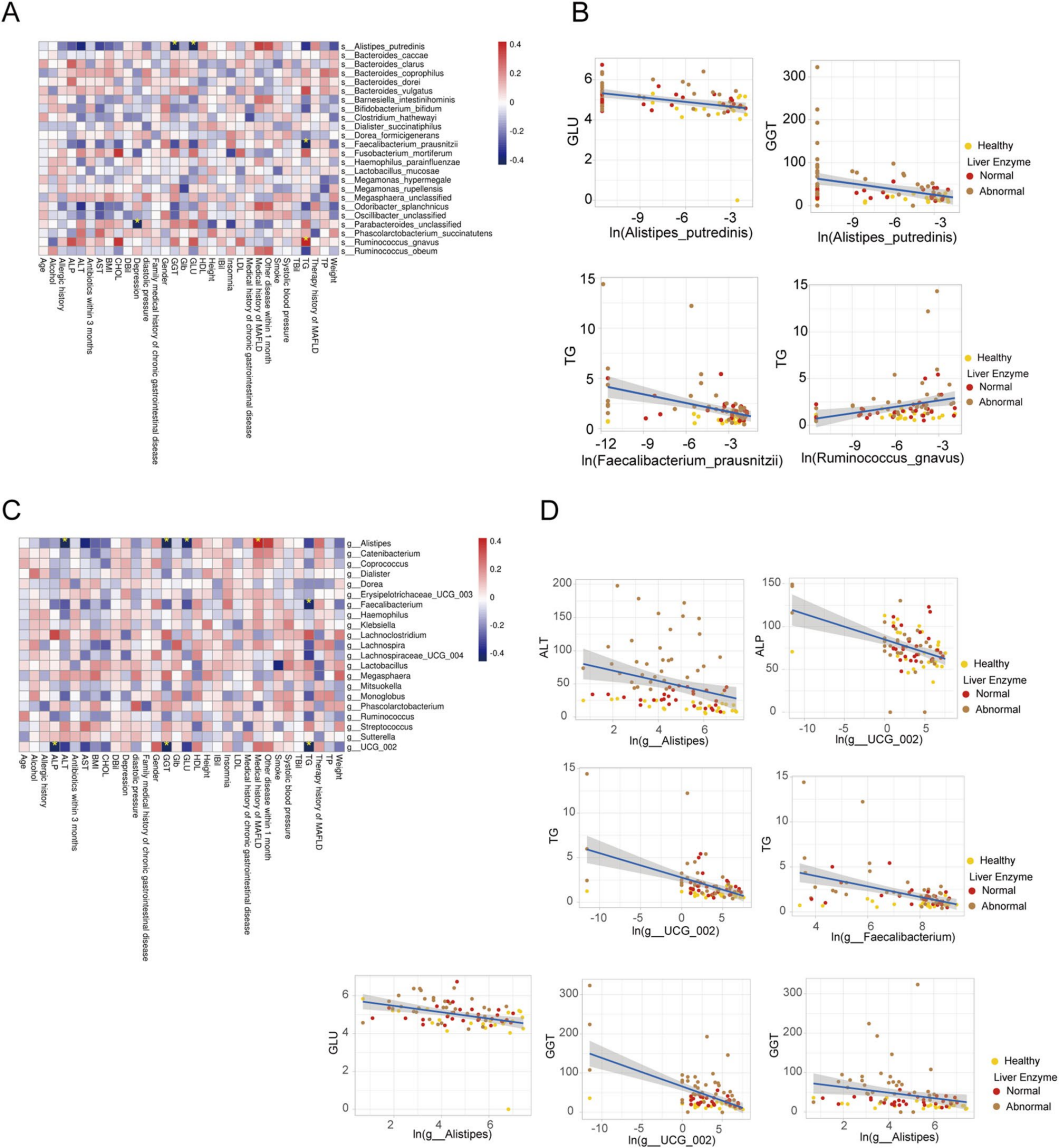
Relationship of gut microbiome and clinical parameters. (**A**) The association between species level bacteria (metagenomics) and clinical variable using spearman correlation. (**B**) The correlation of representative microbiome and clinical parameters in different groups. (**C**) The association between genus level bacteria (16S) and clinical variable using spearman correlation. (**D**) The correlation of representative microbiome and clinical parameters in different groups. *P < 0.05.

To further explore the relationships between disturbances of gut microbiome and clinical variables, spearman correlation analysis was performed. We found that the differential bacterial microbiomes were generally associated with clinical variables (Fig. 6A). Furthermore, the representative microbiome and clinical factors with significant positive or negative correlations (P < 0.05, |Rho|> 0.4) between the indicated groups were shown by the correlation scatter plots as well (Fig. 6B). For example, *Alistipes putredinis* was significantly negatively correlated with GLU and GGT levels, *Faecalibacterium prausnitzii* was negatively correlated with TG, but *Ruminococus gnavus* was positively related to TG between the healthy controls, MAFLD subgroups with abnormal liver enzyme and normal liver enzyme, respectively. Collectively, these results suggested that *Ruminococus gnavus* might promote MAFLD progression. In contrast, *Alistipes putredinis* and *Faecalibacterium prausnitzii* may be useful in the symptomatic relief of MAFLD.

### Associations between KEGG Orthology, genus by 16S sequencing and clinical parameters

Correlation analysis between the KEGG Orthology and clinical variables in both MAFLD and healthy group showed that TG was negatively associated with K00558, K02003 and so on (Fig. S1A,B).

On the other hand, gut microbiome genus with clinical factors had much different patterns of associations (P < 0.05, |Rho|> 0.4). As shown in Fig. 6C,D, *Alistipes* was negatively related to GLU, ALT, and GGT, *UGG_002* was negatively related to ALP, TG, and GGT, *Faecalibacterium* was negatively related to TG.

### Associations between gut microbiome species with the degree of abnormal liver enzymes

We re-analyzed the liver enzymes levels of these patients, GGT, ALT, and AST levels above the upper limit of normal (ULN) were included as three indexes for the abnormal liver enzymes subgroups. Four types of abnormal liver enzymes set as: 1N (1ULN, at least 1 index reached 1xULN AND under 1.5xULN), 1.5N (at least 1 index reached 1.5xULN AND under 2xULN), 2N (at least 1 index reached 2xULN AND under 3xULN), and 3N (at least 1 index reached 3xULN AND above).

**Figure 7:**
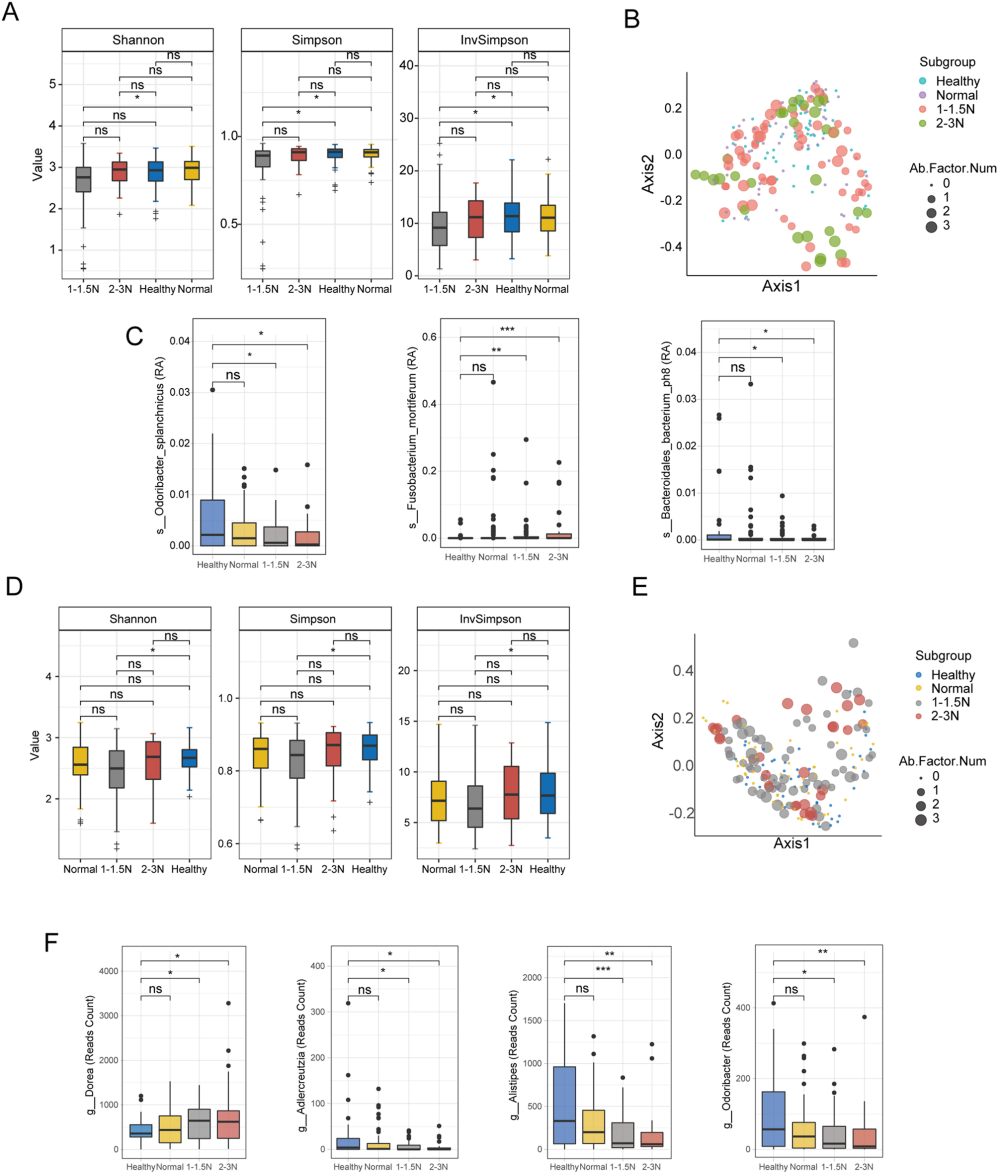
The comparison of gut microbiome with the degree of abnormal liver enzymes. (**A**) Alpha diversity indices of the gut microbiome in different group in species level using metagenomics data. (**B**) The decomposition visualization of the beta diversity among the 4 groups in species level using metagenomics data. (**C**) Relative abundance of differential abundance microbiome species in different groups. (**D**) Alpha Diversity indices of the gut microbiome in different group in genus level using 16S data. (**E**) The decomposition visualization of beta diversity among the 4 groups. (**F**) Relative abundance of differential abundance microbiome genus in different groups. *P < 0.05, **P < 0.01, ***P < 0.001, ns *P* > 0.05.

The gut microbial species α‐diversity analysis revealed that the Shannon, Simpson and InvSimpson diversity indexes were lower in 1–1.5N group than in 2-3N, healthy, and normal liver enzymes groups; however, there was no significant difference between other groups (Fig. 7A). The microbiome compositions between the 1-1.5N and normal groups (PERMANOVA R^2^ = 0.0142, *P* = 0.021), 2-3N and healthy group (PERMANOVA R^2^ = 0.0335, *P* = 0.002) were significantly different (Fig. 7B).

To further investigate key microbiome related to the severity of MAFLD, we performed the relation analysis among microbiome genus and species with disease severity. According to the three subgroups above, including 1-1.5N, 2-3N, and normal liver enzyme group, we calculated the intersection of the three sets and obtain 3 species the 1-1.5N and 2-3N (Fig. 7C). Further analysis revealed that *Odoribacter splanchnicus* and *Bacteroidales bacterium ph8* were significantly enriched within healthy group in contrast to the subgroups of 1-1.5N and 2-3N. Moreover, *Fusobacterium mortiferum* was enriched within the subgroups of 1-1.5N and 2-3N in contrast to healthy group.

### Associations between gut microbiome genus with the degree of abnormal liver enzymes

The gut microbial genus α‐diversity analysis revealed that the Shannon, Simpson and InvSimpson diversity indexes were lower in 1-1.5N subgroup than in healthy groups; however, there was no significant difference between other groups (Fig. 7D). The microbiome composition difference between the MAFLD normal enzyme subgroup and healthy groups (PERMANOVA R^2^ = 0.0188, P = 0.034), 1-1.5N and healthy groups (PERMANOVA R^2^ = 0.0252, P = 0.004), 2-3N and healthy group (PERMANOVA R^2^ = 0.0271 P = 0.039) were significantly different, indicating that there were composition differences between healthy and other groups (Fig. 7E). For gut microbiome genus, we obtained 5 genus from the 1-1.5N and 2-3N (Fig. 7F) subgroups. We found that *Adlercreutzia*, *Alistipes*, and *Odoribacter* were significantly enriched within healthy group in contrast to the 1-1.5N and 2-3N subgroups. *Lachnospiraceae NK4A136* was enriched within healthy group in contrast to the 2-3N subgroup. Of note, *Dorea* was enriched in the 1-1.5N and 2-3N subgroups in contrast to healthy group.

## Discussion

The pathophysiology underlying MAFLD is a complex process that depends on many interconnecting factors, such as insulin resistance, lipotoxicity, infiltration of pro- inflammatory cells, hepatic stellate cell (HSC) fibrogenesis and over-activation^26,27^.

However, to date, no approved pharmacological agents are available for MAFLD treatment. Recent reports have shown that the gut microbiota may promote the progress of MAFLD through the intestinal-hepatic axis pathway^28^. Microbiota can aggravate or improve liver diseases through several mechanisms, including elevated alcohol production, enhanced liver lipid metabolism, impaired intestinal permeability, altered energy metabolism, and disrupted bile secretion^29,30^.

For the purpose of further verifying the detailed microbiome characteristic of patients with MAFLD for developing novel treatment remedies, we took advantage of the metagenomic sequencing and 16S-based sequencing to study the characteristics of the gut microbiome composition in MAFLD patients in China. In current study, we focus on how the gut microbiota promote MAFLD development via the gut-liver axis and explore gut microbiome potential as a novel diagnostic biomarker and therapeutic strategy for MAFLD.

We identified signature microbial taxa within MAFLD patients as well as taxa that were altered among the subgroups of different liver enzyme. Besides, we also found that a trend of depletion for key gut microbial taxa toward abnormal level of liver enzymes, suggesting gut dysbiosis can induce disease progression. On the other hand, the key gut microbial taxa were enriched in the healthy group may have helped to alleviate disease.

For instance, *Ruminococcus obeum* and *Alistipes* were most enriched in healthy individuals. Importantly, loss of *Ruminococcus* has been associated with susceptibility to dextran sodium sulphate–induced colitis^31^. *Alistipes* belongs to the family *Rikenellaceae*, which is also decreased in patients with NAFLD^21^. Moreover, *Alistipes* has demonstrated a good anti-inflammatory effect in human and animal experiments^32^. Several studies have linked the presence of *Alistipes* genus with a healthy state^22^. Also in the present study, it was found that *Alistipes* has negatively related to GLU, GGT, and ALT.

As a result of the functional crosstalk between the liver and the complex microbial composition, microbial imbalance has been identified as a key player in MAFLD's pathogenesis. Our study used MSEA analysis to analyze the intestinal microbiota association with MAFLD. We showed that *Dorea*, *Lactobacillus*, *Megasphaera* are enriched in MAFLD group. Although *Dorea* is thought to be a constituent of healthy gut microflora, it has been linked with inflammatory diseases, such as inflammatory bowel disease (IBD), where patients exhibit an abundance of *Dorea*, suggesting a pro-inflammatory role for this bacterium^33^. Rocha-Ramírez, L. M. et al. have shown that *Lactobacillus* stimulate TNF-α production^34^, therefore, *Lactobacillus* may induce changes in inflammatory factors that induce MAFLD. The genus *Megasphaera* was associated with human papillomavirus (HPV) positive head and neck squamous carcinoma and lung cancer^35,36^. Overweight and obesity are related to the pathophysiology of MAFLD. *Megasphaera* can ferment excess carbohydrates into SCFAs and improve energy absorption. It was more abundant in obese people^37^. We suggest that the gut microbiota is involved in different aspects of the pathogenesis of MAFLD.

Moreover, the abundance of *Dorea* was found to be significantly overrepresented in the MAFLD patients and the degree of enrichment increased with increasing abnormal liver enzyme. These findings indicate that *Dorea* might have potency to induce MAFLD, and implicating the potential of the gut microbial taxa as non-invasive biomarkers for predicts for severity of MAFLD. Velázquez et al. found that *Dorea* levels were significantly elevated in NASH patients^38^. Moreover, *Dorea* level was associated with obesity and increased levels of glutamate^39–41^. Several reports have described associations between a high abundance of *Dorea* and some diseases, such as irritable bowel syndrome and colorectal adenomas^42–44^.

One primary limitation of our study is the modest sample size and therefore limited statistical power to detect subtle effects. We would like to expand the overall sample size in our future research, which may provide the possibility to analyze the association of gut microbiome characteristic with MAFLD patients, especially for the subgroup analysis. Moreover, the results need to be validated in further larger cohorts preferably with different demographic backgrounds. Besides, specific biological experiments are needed to further validation of the target flora. And future studies should also evaluate the significance of these microbial changes for the diagnosis, prognosis, and drug response of MAFLD.

In summary, we showed that the diversity and composition of the microbiome of MAFLD patients differed from those in healthy subjects in China. An increase in *Dorea*, combined with decreases in *Alistipes* appear to be characteristic of MAFLD patients. The roles of these gut Microbiome in the etiology and pathogenesis of MAFLD require further exploration.

## Methods

### Ethics

The study was conducted in accordance with the Declaration of Helsinki (as revised in 2013). Ethical approval for this study was obtained from the Shenzhen Hospital of Southern Medical University Ethic Committee (NYSZYYEC20210019) and clinical study registration was completed (ChiCTR2100051634). Written informed consent was obtained from all study participants. All methods were conducted in accordance with the relevant guidelines and regulations.

### Overall study design

This is a non-interventional, prospective study to explore the gut microbial characteristics in MAFLD patients. All participants must sign informed consent to be included in the study. First 50 MAFLD patients enrolled into the study must meet the enrollment criteria of Stage 1 (for overall analysis. See section of inclusion and exclusion criteria for detail). MAFLD patients enrolled after the first 50 patients, must meet the enrollment criteria of Stage 2 (for overall and subgroup analysis). After study participants were enrolled into the study, 2 stool samples were collected by each study participants from 2 consecutive defecations within a week. Study participation was ended after the completion of stool sample collection.

### Recruitment of subjects and sample collection

One-hundred and thirty-eight (138) MAFLD patients and twenty-eight (28) healthy volunteers were screened according to the inclusion and exclusion criteria in the Shenzhen Hospital of Southern Medical University. Fifty-seven (57) MAFLD patients and three (3) healthy volunteers failed screening. Hence, eighty-one (81) MAFLD patients and twenty-five (25) healthy volunteers were enrolled into the study and were included in the final analysis.

Out of eighty-one (81) MAFLD patients, 10 patients with diabetes were only included in the overall analysis. 34 patients with abnormal liver enzyme (Liver Enzyme Abnormal, LEA group), and 28 patients with normal liver enzyme (Liver Enzyme Normal, LEN) were included in both overall and subgroup analysis. 9 patients with slightly abnormal liver enzyme (Liver Enzyme Slightly Abnormal, LESA) was included only in some of the analysis. Liver enzyme parameters used for dividing patients into the subgroups are GGT, AST, and ALT. For LEA subgroup, the patients had at least one of the three parameters at ≥ 2xULN; the patients who had all abnormal liver enzyme levels at < 2xULN are in the LESA subgroup.

Fecal samples were obtained from all study subjects. Sterile collection bowl and spatula, as well as the collection tube with DNA stabilizing solution were provided to all study participants. At defecation, feces were first collected into the sterile collection bowl, then was spooned into the collection tube. After tightly close the cap, the tube was inverted 5–6 times to mix the DNA stabilizing solution with the stool matters. About 1 g of stool sample was collected. The samples then were sent to the central lab at room temperature, then stored under – 75 °C.

### The inclusion and exclusion criteria

All study participants must sign informed consent. Adults, age of 18–70 years old, who were diagnosed as MAFLD according to the international expert consensus statemen^45^, were enrolled into the study. For Stage 1, MAFLD study participants must have: (1) evidence of hepatic steatosis by hepatic histopathological diagnosis, imaging, or serum biomarkers/NAFLD activity score (NAS); (2) One of the following conditions: a. overweight/obesity (BMI ≥ 23 kg/m^2^); b. presence of type 2 diabetes mellitus (Fasting GLU ≥ 7.0 mmol/L; c. metabolic dysfunction, with at least two of the following conditions: (a) high waist circumference; (b) hypertension; (c) hypertriglyceridemia; (d) high-density lipoprotein-cholesterol; (e) prediabetes; (f) insulin resistance; (g) hyper-sensitivity C-reactive protein level. For Stage 2, MAFLD participants must have: (1) evidence of hepatic steatosis by hepatic histopathological diagnosis, imaging, or serum biomarkers/ NAFLD activity score (NAS); (2) overweight or obesity (BMI ≥ 23 kg/m^2^).

The exclusion criteria were: (1) other chronic disorders, including but not limited to chronic infection, apparent intestinal illness or symptoms (including but not limited to constipation, diarrhea, ulcerative colitis, Crohn’s disease, bowel obstruction, and intestinal malignant tumors); (2) other severe acute diseases 1 month prior to the screening visit that may interfere with data analysis; (3) cirrhosis; (4) (for Stage 2 only) with type 2 diabetes or metabolic dysfunction; (5) Participants who have taken oral antibiotics 1 month prior to the screening visit; (6) within 1 week of sample collection, excessive alcohol consumption (> 1000 mL of beer or > 200 mL of Chinese Baijiu), or sudden and significant change in diet; (7) any conditions such as malaise, poor compliance, etc. that PI considered not suitable for this study.

### DNA library construction and metagenomic sequencing

Fecal DNA were extracted by following the protocol in DNeasy® PowerSoil® Pro Kit Handbook (Catalog No. 47016) and were stored at -20℃. KAPA Hifi HotStart ReadyMix and KAPA Hyper Prep Kit (KR0961–v4.15) were used for library construction for Amplicon (16S V4 region) sequencing and metagenomics sequencing respectively.

### 16S rRNA gene amplification sequencing and shotgun metagenomic sequencing

Novaseq 6000 was used to sequence both 16S and metagenomics libraries. The extracted fecal DNA was used as the template for amplicon sequencing with barcoded primers 515F: 5ʹ-GTGCCAGCMGCCGCGGTAA-3ʹ and 805R: 5ʹ-GACTACNVGGGTATCTAATCC-3ʹ flanking the V4 hypervariable region of the nectarial 16S rRNA gene.

### Taxonomic profiling and functional profiling

Raw reads from all samples were quality filtered and denoised with DADA2^19^ to generate amplicon sequencing variants (ASV). Taxonomy assignment of each ASV was accomplished by DADA2 with the SILVA^46^ v138 reference database and the reads profile at Genus level was extracted. Genus with read count less than 10 in each sample were aggregated as low abundance taxonomies. Raw reads from metagenomic sequencing were quality controlled by fastp^47^ (version 0.23.1). After trimming the low-quality portion of reads and subsequently removing reads shorter than 50 bp, we used bowtie2^48^ to map the filtered reads to the human genome (hg19) to remove host contaminants. Remaining reads were fed to MetaPhlAn2^49^ (version 2.7.5) for taxonomy profiling with default parameters. Functional analysis was conducted with HUMAnN2^24^ (version 2.8.1) by aligning clean reads (reads after removing host contamination) to KEGG protein database (www.kegg.jp/kegg/kegg1.html) (version 2020.7)^50^. Furthermore, mapped KEGG genes were enriched to KO entries by MinPath^25^.

### Microbe-set enrichment analysis (MSEA)

MSEA (Microbe-set enrichment analysis) is a useful knowledge-based computational approach to interpret the functions of microbes, which can be integrated with microbiome profiling pipelines to help reveal the underlying mechanism of host-microbiome interactions. We conducted MSEA on differentially abundant Genus screened from LEfSe^51^ analysis and enriched the most significant (top 10) human genes to KEGG pathways by EnrichR package in R.

### Statistical analyses

All analyses of microbiome were done in genus level with 16S data and in species level with metagenomics data. Statistics was performed using R software 3.6.3. The alpha diversity was estimated by species richness from the Shannon, Simpson, and InvSimpson index metrics. The beta diversity was estimated by computing bray–curtis distances and was visualized with principal coordinate analysis^52^. PERMANOVA using “MASS” and “Vegan” in R package were used to identify significant differences in distance between different groups. To identify the significant different species, the linear discriminant analysis (LDA) effective size (LEfSe)^51^ was conducted. Weighted and unweighted UniFrac distances were used for Principal Coordinates Analyses (PCoA). The Kyoto Encyclopedia of Genes and Genomes (KEGG) pathways for the functional analysis of gene sequences. Two-sided Wilcoxon’s rank-sum test was used for c assess differences in alpha and beta diversity of the two groups. The Kruskal–Wallis test was used for comparisons among multi-groups. Fisher exact test was applied to check the dependency of categorical variables. The Spearman’s rank test was performed for correlation analysis. *P* values of 0.05 or less were considered statistically significant and P values for Fisher exact test and Wilcoxon test were adjusted with FDR method with significant level set to 0.05.

## Supplementary Information

Supplementary Information.

## Data Availability

The 16S rRNA gene sequencing and metagenomic sequencing read for this study are available in the NCBI SRA database as project accessions PRJNA930148 and PRJNA924942.
