# Supplementary material for: Characteristics of gut microbiota in patients with metabolic associated fatty liver disease
Source: Sci Rep. 2023 Jun 20;13:9988. doi: 10.1038/s41598-023-37163-4 (PMC10281992; doi:10.1038/s41598-023-37163-4)
Supplement: Supplementary file 1 — Supplementary Information. [file 41598_2023_37163_MOESM1_ESM.pdf]

A

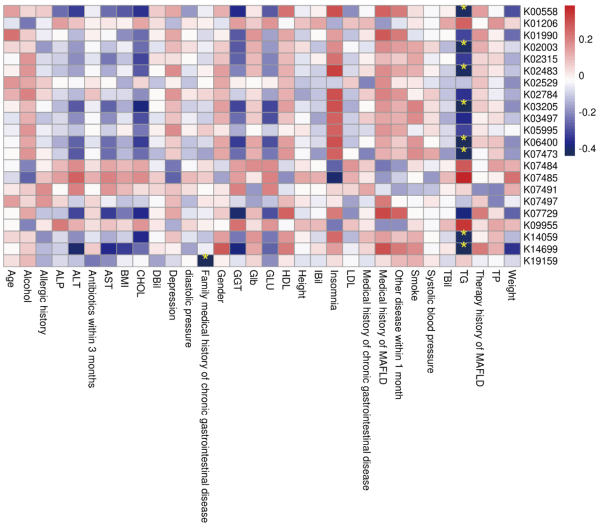

B

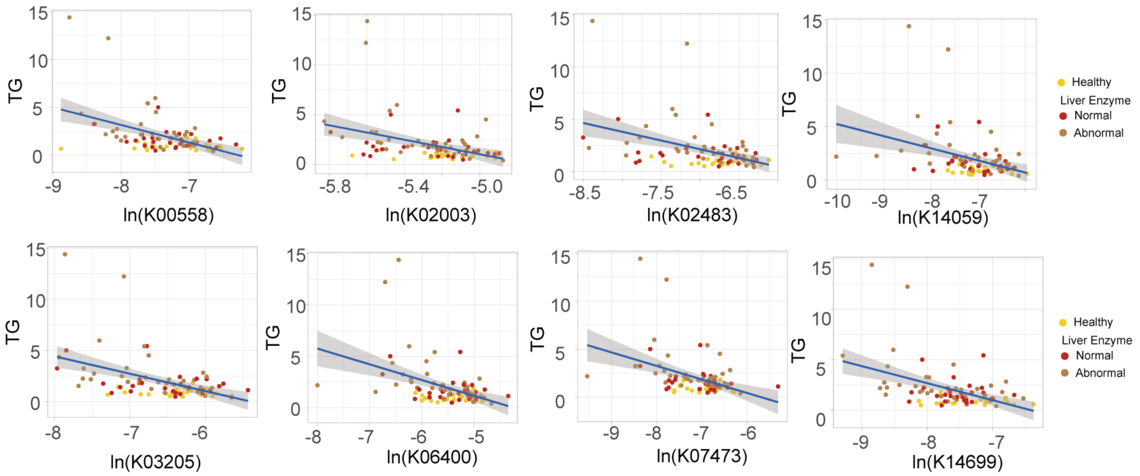

Healthy
Liver Enzyme
Normal
Abnormal
TG
ln(K00558)
ln(K02003)
ln(K02483)
ln(K14059)
ln(K03205)
ln(K06400)
ln(K07473)
ln(K14699)

Figure S1. KEGG Orthology and clinical variables.

A. Heatmap shows the association between the KEGG Orthology and clinical variables.

B. Correlation scatter plots showed the correlation of representative pathway and clinical parameters in different groups. *$P < 0.05$.

Supplementary table 1. Beta dispersion test and PERMANOVA test of gender in species level using metagenomics data

	SumsOfSample	Df	SumSq	MeaSq	F	R2	Pr(>F)
**Beta dispersion**	81	1	0.01	0.01	0.82		0.38
**Permanova**	81	1	0.33	0.33	1.12	0.01	0.30

Supplementary table 2. Beta dispersion test and PERMANOVA test of gender in genus level using 16S data

	SumsOfSample	Df	SumSq	MeaSq	F	R2	Pr(>F)
**Beta dispersion**	81	1	0.00	0.00	0.00		0.97
**Permanova**	81	1	0.14	0.14	0.88	0.01	0.46

Supplementary table 3. Beta dispersion test and PERMANOVA test of age in species level using metagenomics data

	SumsOfSample	Df	SumSq	MeaSq	F	R2	Pr(>F)
**Beta dispersion**	81	1	0.00	0.00	0.44		0.53
**Permanova**	81	1	0.49	0.49	1.68	0.02	0.04

Supplementary table 4. Beta dispersion test and PERMANOVA test of age in genus level using 16S data

	SumsOfSample	Df	SumSq	MeaSq	F	R2	Pr(>F)
**Beta dispersion**	81	1	0.00	0.00	0.12		0.71
**Permanova**	81	1	0.26	0.26	1.72	0.02	0.09

Supplementary table 5. Summary of medication used by liver enzyme abnormal (LEA) and liver enzyme normal (LEN) patients

	LEA (n=34)	LEN (n=28)	p-value (adjust.pvalue)
**mafld**	28/34 (82%)	7/28 (25%)	0.00(0.00)
**lipid lower**	8/34 (24%)	3/28(11%)	0.32(0.43)
**gi**	4/34 (12%)	2/28 (7%)	0.68(0.68)
**others**	5/34 (15%)	1/28 (3.5%)	0.21(0.42)
